# Supplementary material for: Visible quantum plasmonics from metallic nanodimers
Source: Sci Rep. 2016 Oct 18;6:34772. doi: 10.1038/srep34772 (PMC5067502; doi:10.1038/srep34772)
Supplement: Supplementary Information [file srep34772-s1.pdf]

Supplementary Information:
Visible quantum plasmonics from metallic nanodimers

F. Alpeggiani,[1,*] S. D'Agostino,[2] D. Sanvitto,[3] and D. Gerace[1,†]
[1] *Dipartimento di Fisica, Università di Pavia, via Bassi 6, 27100 Pavia, Italy*
[2] *Center for Biomolecular Nanotechnologies @ UNILE - Istituto Italiano di Tecnologia, 73010 Arnesano, Italy*
[3] *CNR NANOTEC - Institute of Nanotechnology, Via Monteroni, 73100 Lecce, Italy*

COMPUTING QUASINORMAL MODES

We hereby give the detailed procedure employed to compute the characteristics of localized surface plasmons in metallic dimers. Surface plasmons are identified with quasi-normal modes (QNMs), i.e., the solutions of a non-Hermitian electromagnetic problem, which are calculated in the context of the boundary element method (BEM) described in Ref. 1. All calculations are performed within the MNPBEM toolbox [2, 3], which constitutes a freely available implementation of the BEM.

According to the BEM formalism of Ref. 1, space is subdivided in several regions V_j $(j = 1, 2, \dots)$ with locally homogeneous dielectric functions (in our case, the inner region of the dimer and the external space). The electric field in each region is decomposed into an incident and a scattered part, the latter being identified with the field generated by (fictitious) charge and current distributions $\sigma_j(\mathbf{r})$ and $\mathbf{h}_j(\mathbf{r})$ on the enclosing surface, as follows:

$$\mathbf{E}_{\rm sc}(\mathbf{r},\omega) = i\frac{\omega}{c}\int_{\partial\Omega_j} G_j(\mathbf{r}-\mathbf{s})\mathbf{h}_j(\mathbf{s})\,\mathrm{d}\mathbf{s} - \nabla\int_{\partial\Omega_j} G_j(\mathbf{r}-\mathbf{s})\sigma_j(\mathbf{s})\,\mathrm{d}\mathbf{s}. \tag{1}$$

$G_j(\mathbf{r}) = \exp(ik_j|\mathbf{r}|)/|\mathbf{r}|$ is the scalar Green function for region j. Distributions $\sigma_j(\mathbf{r})$ and $\mathbf{h}_j(\mathbf{r})$ are the unknowns of the problem, and they must be computed from boundary conditions at the separating interfaces.

After the surface is discretized into a set of N simpler polygons (triangles and quadrilaterals), surface charge and current distributions become N-dimensional vectors. As illustrated in the text, the QNMs of the system are obtained by solving the nonlinear eigenvalue problem [4]

$$\Sigma(\tilde{\omega})\,\tilde{\mathbf{x}} = 0 \tag{2}$$

for the (complex) eigenvalue $\tilde{\omega}$ and the right eigenvector $\tilde{\mathbf{x}}$. The matrix Σ is defined after Eq. (22) of Ref. 1. Once the eigenvector $\tilde{\mathbf{x}}$ is known, the corresponding charge and current distributions are obtained from the following expressions:

$$\begin{aligned}\sigma_j &= G_j^{-1}\,\tilde{\mathbf{x}},\\ \mathbf{h}_j &= i\frac{\omega}{c}\hat{\mathbf{n}}\,G_j^{-1}\Delta^{-1}(L_1 - L_2)\,\tilde{\mathbf{x}},\end{aligned} \tag{3}$$

where $j = 1, 2$ indicates the inner and outer regions of the particle, respectively, $\hat{\mathbf{n}}$ is the normal vector to each surface element, and we still refer to Ref. 1 for the definition of the matrices G_j, L_j, and Δ. The quasinormal field $\mathcal{E}(\mathbf{r})$ is calculated (up to a normalization constant that will be fixed in the following) by replacing the charge and current distributions of Eqs. (3) into Eq. (1).

We have solved the nonlinear eigenproblem (2) with the two-sided Rayleigh functional iterative algorithm [4], which allows to compute the eigenvalue and the right and left eigenvectors, indicated as $\tilde{\mathbf{x}}$ and $\tilde{\mathbf{y}}$, respectively. Starting from an initial triplet $(\tilde{\mathbf{x}}_0, \tilde{\omega}_0, \tilde{\mathbf{y}}_0)$, at each iteration k the values of the three quantities is updated with respect to the previous estimate by solving the equations:

$$\Sigma(\tilde{\omega}_k)\,\mathbf{x}_{k+1} = \Sigma'(\tilde{\omega}_k)\,\mathbf{x}_k; \tag{4}$$

$$[\Sigma(\tilde{\omega}_k)]^*\,\mathbf{y}_{k+1} = [\Sigma'(\tilde{\omega}_k)]^*\,\mathbf{y}_k; \tag{5}$$

$$\tilde{\omega}_{k+1} = \tilde{\omega}_k - \frac{\mathbf{y}_{k+1}^*\,\Sigma(\tilde{\omega}_k)\,\mathbf{x}_{k+1}}{\mathbf{y}_{k+1}^*\,\Sigma'(\tilde{\omega}_k)\,\mathbf{x}_{k+1}}. \tag{6}$$

The notation Σ' indicates the derivative $d\Sigma/d\omega$, which can be approximated by finite differences by storing the values of the previous iteration, whereas Σ^* indicates the conjugate transpose. In order to speed up convergence, the initial guess $\tilde{\mathbf{x}}_0$ can be extracted from the solution of the electromagnetic problem with a suitable external excitation (e.g.,

a dipole emitter) near resonance. In addition, the vectors $\tilde{\mathbf{x}}$ and $\tilde{\mathbf{y}}$ can be renormalized at each iteration to reduce overflow errors. As the eigenfrequency $\tilde{\omega}$ is typically complex, we had to modify the MNPBEM toolbox to extend the refractive index of the particle to the complex domain, by means of a first-order analytical continuation of the form

$$n_{\mathrm{met}}(\omega' + i\omega'') \approx n_{\mathrm{exp}}(\omega') + i\omega'' \frac{dn_{\mathrm{exp}}(\omega')}{d\omega'}, \tag{7}$$

where n_{exp} is a cubic spline interpolation of experimental data and the derivative of the resulting piecewise polynomial is calculated analytically. As stated in the text, we employed the tabulation of the experimental dielectric function of evaporated gold published in Ref. 5.

In order to ensure the proper normalization of the QNM, we follow an implicit approach involving an additional dipole source, similar to that presented in Ref. 6. We add a point dipole source with momentum $\mathbf{p} = p\hat{\mathbf{x}}$ at the position $\mathbf{r}_0$ in the center of the dimer gap (see the inset in Fig. 2 of the main text) and we calculate the field scattered back by the particle to the dipole for $\omega \to \tilde{\omega}$. The normalization condition, reported by Eq. (2) of the main text, reads as follows:

$$\mathbf{E}_{\mathrm{sc}}(\mathbf{r}, \omega) \approx -\omega \frac{\mathbf{p} \cdot \mathcal{E}(\mathbf{r}_0)}{2\varepsilon_0(\omega - \tilde{\omega})} \mathcal{E}(\mathbf{r}). \tag{8}$$

Notice that both terms are proportional to the momentum $\mathbf{p}$. The dependence in the left-hand term is implicit. The condition can be simplified by replacing the matrix Σ^{-1} in the calculation of the scattered field with the polar approximation for $\omega \approx \tilde{\omega}$ [4]

$$\Sigma^{-1}(\omega) \approx \frac{1}{\omega - \tilde{\omega}} \frac{\tilde{\mathbf{x}}\tilde{\mathbf{y}}^*}{\tilde{\mathbf{y}}^*[d\Sigma/d\omega]\tilde{\mathbf{x}}}, \tag{9}$$

where $\tilde{\mathbf{x}}$ and $\tilde{\mathbf{y}}$ are the right and left eigenvectors, respectively. This allows to factor out the singular term from both sides of Eq. (8) and to recast the normalization condition in the form

$$\mathbf{p} \cdot \mathcal{E}(\mathbf{r}_0) = -\frac{2\varepsilon_0}{\tilde{\omega}} \frac{1}{\tilde{\mathbf{y}}^*[d\Sigma/d\omega]\tilde{\mathbf{x}}} \tilde{\mathbf{y}}^* \Big\{ D^e - \Sigma_1 L_1 \phi^e + i\frac{\omega}{c}\hat{\mathbf{n}} \cdot \left[(L_1 - L_2)\Delta^{-1}(\alpha + ik\hat{\mathbf{n}}L_1\phi^e) + (L_2\Delta^{-1}\Sigma_1 - L_1\Delta^{-1}\Sigma_2)\mathbf{A}^e \right] \Big\}. \tag{10}$$

The term in curly brackets is the same as that in the right-hand side of Eq. (22) in Ref. 1 (where the definition of all included quantities can be found), and it is a source term constructed from the field emitted by the dipole.

Equation (8) does not require any further calculations than those already performed in order to solve the nonlinear eigenproblem, greatly improving the computational efficiency. We employed surface meshes with up to about 6000 elements. Typically, a few iterations of the algorithm are enough to converge to the solution of the eigenproblem. The computational time for the calculation of a single QNM (including normalization) is of the order of a few minutes with a quad-core 3.1 GHz personal computer.

The MNPBEM toolbox has been employed also to calculate the semiclassical decay rate of the dipole emitter of Fig. 2(a) of the main text. The rate is computed through the formula [7]:

$$\Gamma(\omega) = \Gamma_{\mathrm{free}}(\omega) + \frac{2}{\hbar} \mathrm{Im}\left[\mathbf{p} \cdot \mathbf{E}_{\mathrm{sc}}(\mathbf{r}_0)\right], \tag{11}$$

where $\mathbf{E}_{\mathrm{sc}}(\mathbf{r}_0)$ is the field scattered back by the nanodimer at the dipole position.

DISCRETE DIPOLE APPROXIMATION

The modification of the dipole emitter total decay rate induced by the presence of the dimer has also been quantitatively assessed in the framework of the discrete dipole approximation (DDA) full-wave simulation method, which describes the scatterer as an array of polarizable points organized on a regular cubic grid [8]. This method yields solutions for the electromagnetic field in response to an incident electric field in the frequency domain, including retardation effects. The polarization of each element internal to the scatterer is seen as the result of the interaction with the local electromagnetic field produced by all the other dipoles plus the external field of the emitter. By simply fixing the dipole position in space, the changes induced by the dimer antenna on the local field experienced by the dipole can be rigorously calculated. The validity of the method been largely tested in recent works [9].

The simulations for the present work have been performed by using the parallel code ADDA in its final version, which implements dipolar sources [10]. The inter-dipole distance is fixed to 0.25 nm - a value that is small enough to achieve numerical convergence - and the gold refractive index is taken from Ref. 5, similarly to the BEM calculations described before.

QUANTIZATION PROCEDURE FOR QUASINORMAL MODES

In the context of the system-bath approach for the quantization of the electromagnetic field in the presence of dispersive and dissipative media, the electric field operator is expanded on a continuum of “true” normal modes including the effect of the dispersive media, in the following form [11, 12]:

$$\hat{\mathbf{E}}(\mathbf{r}) = i\sqrt{\frac{\hbar}{\pi\varepsilon_0}}\int \mathrm{d}\omega\,\frac{\omega^2}{c^2}\int \mathrm{d}\mathbf{r}'\sqrt{\mathrm{Im}\,\varepsilon(\mathbf{r}',\omega)}\,\overleftrightarrow{G}(\mathbf{r},\mathbf{r}',\omega)\hat{\mathbf{f}}(\mathbf{r}',\omega) + \mathrm{h.c.}\,, \tag{12}$$

where $\hat{\mathbf{f}}(\mathbf{r},\omega)$ are (vector) destruction operators satisfying the bosonic commutation relation $[\hat{f}_j(\mathbf{r},\omega),\hat{f}_k^\dagger(\mathbf{r}',\omega')] = \delta(\mathbf{r}-\mathbf{r}')\delta(\omega-\omega')\delta_{jk}$ $(j,k=1,2,3)$, and “h.c.” indicates the Hermitian conjugate operator. The quantity $\overleftrightarrow{G}(\mathbf{r},\mathbf{r}',\omega)$ is the dyadic Green function of the (classical) electric field, satisfying $\nabla\times\nabla\times\overleftrightarrow{G}(\mathbf{r},\mathbf{r}',\omega) - \omega^2\varepsilon(\omega)\overleftrightarrow{G}(\mathbf{r},\mathbf{r}',\omega)/c^2 = \overleftrightarrow{I}\delta(\mathbf{r}-\mathbf{r}')$. Each tensor component G_{jk} is defined as the j-component of the electric field at point $\mathbf{r}$ calculated in the presence of the dispersive media for a point-like current source at point $\mathbf{r}'$ and of the form: $\mathbf{J}(\mathbf{r}) = -i\delta(\mathbf{r}-\mathbf{r}')\hat{\mathbf{x}}_k/(\omega\mu_0)$. The source corresponds to an oscillating dipole with momentum $\mathbf{p} = \hat{\mathbf{x}}_k/(\omega^2\mu_0)$; as a consequence, we can use the result in Eq. (8) to obtain the expansion of the dyadic Green function in proximity to a QNM:

$$G_{jk}(\mathbf{r},\mathbf{r}',\omega) = -\frac{\omega}{2(\omega-\tilde{\omega})}\frac{c^2}{\omega^2}\mathcal{E}_j(\mathbf{r})\mathcal{E}_k(\mathbf{r}') + G_{\mathrm{other}}(\mathbf{r},\mathbf{r}',\omega). \tag{13}$$

The term $G_{\mathrm{other}}(\mathbf{r},\mathbf{r}',\omega)$ includes the free-space contribution and those of the other QNMs of the system. Since, for $\omega \simeq \mathrm{Re}(\tilde{\omega})$ the dominant term is the pole corresponding to the QNM, we will assume $G_{\mathrm{other}}(\mathbf{r},\mathbf{r}',\omega) \simeq 0$. This assumption is further confirmed by the comparison of the decay rates in Fig. 2 of the main text.

Along the lines of the approach presented in Appendix A.2 of Ref. 12, we introduce the collective modes $\hat{p}(\omega)$ according to the following relation:

$$\mathbf{g}(\mathbf{r},\omega)\hat{p}(\omega) = \sqrt{\frac{\hbar}{\pi\varepsilon_0}}\frac{\omega^2}{c^2}\int \mathrm{d}\mathbf{r}'\sqrt{\mathrm{Im}\,\varepsilon(\mathbf{r}',\omega)}\,\overleftrightarrow{G}(\mathbf{r},\mathbf{r}',\omega)\hat{\mathbf{f}}(\mathbf{r}',\omega). \tag{14}$$

The modes form a continuum depending only on frequency and satisfy the bosonic commutation relation $[\hat{p}(\omega),\hat{p}^\dagger(\omega')] = \delta(\omega-\omega')$. Moreover, we assume the vectors $\mathbf{g}(\mathbf{r},\omega)$ to be real. These assumptions will be justified at the end of the procedure. Following the calculations in Ref. 12, by replacing Eq. (14) into the commutation relation, we obtain:

$$g_j(\mathbf{r},\omega)g_k(\mathbf{r}',\omega) = \frac{\hbar}{\pi\varepsilon_0}\frac{\omega^2}{c^2}\mathrm{Im}G_{jk}(\mathbf{r},\mathbf{r}',\omega). \tag{15}$$

Then, by further replacing the expansion of Eq. (13) into the latter expression, we arrive at

$$g_j(\mathbf{r},\omega)g_k(\mathbf{r}',\omega) = -\frac{\hbar\omega}{2\pi\varepsilon_0}\mathrm{Im}\left[\frac{\mathcal{E}_j(\mathbf{r})\mathcal{E}_k(\mathbf{r}')}{\omega-\tilde{\omega}}\right] \simeq \frac{1}{2\pi}\frac{\hbar\omega_0}{2\varepsilon_0}\left[\frac{\gamma_0}{(\omega-\omega_0)^2+\gamma_0^2/4}\right]\mathcal{E}_j(\mathbf{r})\mathcal{E}_k(\mathbf{r}') \tag{16}$$

$(\tilde{\omega} = \omega_0 - i\gamma_0/2)$. As a result, indicating as $g^2(\omega)$ the quantity in square brackets in the last term, we can write

$$\mathbf{g}(\mathbf{r},\omega) = \sqrt{\frac{\hbar\omega_0}{2\varepsilon_0}}\frac{g(\omega)}{\sqrt{2\pi}}\mathcal{E}(\mathbf{r}), \tag{17}$$

which, together with Eqs. (12) and (14), gives Eq. (3) in the main text. In Eq. (16) we suppose that the QNM field $\mathcal{E}(\mathbf{r})$ can be taken approximately as real, as we verified to be the case for all the systems under consideration in this work ($\mathrm{Re}\mathcal{E} \gg \mathrm{Im}\mathcal{E}$ near the gap region). In other systems, this approximation might not hold. This latter situation corresponds to the case of a non-Lorentzian density of states discussed in Ref. 13, which leads to a pathological master equation not in the Lindblad form considered here.

CALCULATING THE SECOND-ORDER CORRELATIONS

For a given quantized field described by destruction (creation) operators $\hat{A}$ ($\hat{A}^\dagger$), the figure of merit quantifying the single-photon sensitivity is the time-ordered second-order autocorrelation function, defined as [14]

$$G_i^{(2)}(t,t') = \langle \hat{A}^\dagger(t)\hat{A}^\dagger(t')\hat{A}(t')\hat{A}(t)\rangle \,, \tag{18}$$

where $t' - t = \tau > 0$, which is normalized as

$$g^{(2)}(t,t') = \frac{G^{(2)}(t,t')}{\langle \hat{A}^\dagger(t)\hat{A}(t)\rangle\langle \hat{A}^\dagger(t')\hat{A}(t')\rangle} \,. \tag{19}$$

In the manuscript, we have provided results for the numerical calculations of the latter quantities as follows. The master equation in Eq. (5) of the main text is recast in the form

$$\frac{\mathrm{d}\hat{\rho}}{\mathrm{d}t} = \frac{i}{\hbar}[\hat{\rho}, \hat{H}_{\mathrm{rot}}] + \frac{\gamma}{2}\left[2\hat{A}\hat{\rho}\hat{A}^\dagger - \hat{A}^\dagger\hat{A}\hat{\rho} - \hat{\rho}\hat{A}^\dagger\hat{A}\right] \,, \tag{20}$$

where the Hamiltonian is rotated with respect to the driving laser frequency and it is represented as

$$\hat{H}_{\mathrm{rot}} = \hbar\Delta\, \hat{A}^\dagger\hat{A} + U\,(\hat{A}^\dagger)^2\hat{A}^2 + F(t)\hat{A}^\dagger + F^*(t)\hat{A} \,, \tag{21}$$

with $\Delta = \omega_0 - \omega_p$. The Hilbert space is truncated to 11 plasmons, largely sufficient for convergence under the weak pump amplitudes assumed in this work ($F_0/\hbar = 0.01\gamma_0$).

Under continuous wave (cw) excitation, $F(t) = F_0$, the steady state zero-time delay second order correlation can be easily calculated as

$$g^{(2)}(0) = \langle(\hat{A}^\dagger)^2\hat{A}^2\rangle/\langle\hat{A}^\dagger\hat{A}\rangle^2 = \mathrm{Tr}\{(\hat{A}^\dagger)^2\hat{A}^2\hat{\rho}_{ss}\}/n^2 \,, \tag{22}$$

where $n = \mathrm{Tr}\{\hat{A}^\dagger\hat{A}\hat{\rho}_{ss}\}$, and $\hat{\rho}_{ss}$ is the steady state solution corresponding to $\mathrm{d}\hat{\rho}/\mathrm{d}t = 0$.

Under pulsed excitation, e.g. for a train of Gaussian pulses described by $F(t) = F_0\exp\{-(t-nT_0)^2/\Delta T^2\}$ (with $n = 0, \pm1, ...$), where T_0 and ΔT are the pulse separation and width, respectively, Eq. (18) is evaluated numerically through a superoperator evolution [15–17]

$$G^{(2)}(t,t') = \mathrm{Tr}\{\hat{A}\,\mathcal{U}_{t,t'}[\hat{A}\hat{\rho}(t)\hat{A}^\dagger]\hat{A}^\dagger\} \,, \tag{23}$$

where $\mathcal{U}_{t,t'}$ indicates the evolution from t to t' with Eq. 20 when assuming the starting time operator as $\hat{A}\hat{\rho}(t)\hat{A}^\dagger$.

---

* Present address: FOM Institute AMOLF, Science Park 104, 1098 XG Amsterdam, The Netherlands.
† dario.gerace@unipv.it

[1] F. J. Garcia de Abajo and A. Howie, Phys. Rev. B **65**, 115418 (2002)
[2] U. Hohenester and A. Trügler, Comp. Phys. Commun. **183**, 370 (2012).
[3] We used the version MNPBEM13, available online at the web address: `http://physik.uni-graz.at/~uxh/mnpbem/mnpbem.html`
[4] H. Voss, in *Handbook of Linear Algebra*, 2nd ed., edited by L. Hogben (CRC, Boca Raton, Florida, 2014), Chap. 60.
[5] R. L. Olmon, B. Slovick, T. W. Johnson, D. Shelton, S.-H. Oh, G. D. Boreman, and M. B. Raschke, Phys. Rev. B **86**, 235147 (2012).
[6] Q. Bai, M. Perrin, C. Sauvan, J.-P. Hugonin, and P. Lalanne, Opt. Express **21**, 27371 (2013).
[7] U. Hohenester and A. Trugler, IEEE J. Select. Topics Quantum Electron. **14**, 1430 (2008).
[8] S. D'Agostino, F. Della Sala, and L.C. Andreani, Nanomaterials and Nanotechnology **5**, 1 (2015).
[9] S. D'Agostino, F. Della Sala, and L.C. Andreani, Phys. Rev. B **87**, 205413 (2013).
[10] M.A. Yurkin, A.G. Hoekstra, J. Quant. Spectrosc. Radiat. Transfer **112**, 2234 (2011). The code is available online at: `http://code.google.com/p/a-dda/`.
[11] L. Knöll, S. Scheel, and D. Welsch, in *Coherence and Statistics of Photons and Atoms*, edited by J. Perina (Wiley, New York, 2001).
[12] T. Hümmer, F. J. García-Vidal, L. Martín-Moreno, and D. Zueco, Phys. Rev. B **87**, 115419 (2013).
[13] B. M. Garraway, Phys. Rev. A **55**, 2290 (1997).
[14] M. J. Werner and A. Imamoğlu, Phys. Rev. A **61**, 011801(R) (1999).
[15] A. Kiraz, M. Atatüre, and A. Imamoğlu, Phys. Rev. A **69**, 032305 (2004).
[16] A. Verger, C. Ciuti, and I. Carusotto, Phys. Rev. B **73**, 193306 (2006).
[17] A. Majumdar and D. Gerace, Phys. Rev. B **87**, 235319 (2013).
